# Feasibility and Effectiveness of a Web-Based Positive Psychology Program for Youth Mental Health: Randomized Controlled Trial

**DOI:** 10.2196/jmir.3176

**Published:** 2014-06-04

**Authors:** Vijaya Manicavasagar, Deserae Horswood, Rowan Burckhardt, Alistair Lum, Dusan Hadzi-Pavlovic, Gordon Parker

**Affiliations:** ^1^Black Dog InstituteSchool of PsychiatryUNSW AustraliaRandwickAustralia; ^2^School of PsychiatryUNSW AustraliaRandwickAustralia

**Keywords:** adolescent, resilience, psychological, mental health, Internet, early medical intervention

## Abstract

**Background:**

Youth mental health is a significant public health concern due to the high prevalence of mental health problems in this population and the low rate of those affected seeking help. While it is increasingly recognized that prevention is better than cure, most youth prevention programs have utilized interventions based on clinical treatments (eg, cognitive behavioral therapy) with inconsistent results.

**Objective:**

This study explores the feasibility of the online delivery of a youth positive psychology program, Bite Back, to improve the well-being and mental health outcomes of Australian youth. Further aims were to examine rates of adherence and attrition, and to investigate the program’s acceptability.

**Methods:**

Participants (N=235) aged 12-18 years were randomly assigned to either of two conditions: Bite Back (n=120) or control websites (n=115). The Bite Back website comprised interactive exercises and information across a variety of positive psychology domains; the control condition was assigned to neutral entertainment-based websites that contained no psychology information. Participants in both groups were instructed to use their allocated website for 6 consecutive weeks. Participants were assessed pre- and postintervention on the Depression Anxiety Stress Scale-Short form (DASS-21) and the Short Warwick-Edinburgh Mental Well-Being Scale (SWEMWBS).

**Results:**

Of the 235 randomized participants, 154 (65.5%) completed baseline and post measures after 6 weeks. Completers and dropouts were equivalent in demographics, the SWEMWBS, and the depression and anxiety subscales of the DASS-21, but dropouts reported significantly higher levels of stress than completers. There were no differences between the Bite Back and control conditions at baseline on demographic variables, DASS-21, or SWEMWBS scores. Qualitative data indicated that 49 of 61 Bite Back users (79%) reported positive experiences using the website and 55 (89%) agreed they would continue to use it after study completion. Compared to the control condition, participants in the Bite Back condition with high levels of adherence (usage of the website for 30 minutes or more per week) reported significant decreases in depression and stress and improvements in well-being. Bite Back users who visited the site more frequently (≥3 times per week) reported significant decreases in depression and anxiety and improvements in well-being. No significant improvements were found among Bite Back users who demonstrated low levels of adherence or who used the website less frequently.

**Conclusions:**

Results suggest that using an online positive psychology program can decrease symptoms of psychopathology and increase well-being in young people, especially for those who use the website for 30 minutes or longer per week or more frequently (≥3 times per week). Acceptability of the Bite Back website was high. These findings are encouraging and suggest that the online delivery of positive psychology programs may be an alternate way to address mental health issues and improve youth well-being nationally.

**Trial Registration:**

Australian New Zealand Clinical Trials Registry: ACTRN1261200057831; https://www.anzctr.org.au/Trial/Registration/TrialReview.aspx?id=362489 (Archived by Webcite at http://www.webcitation.org/6NXmjwfAy).

## Introduction

### Background

 Addressing mental health problems in young people is a major public health concern [1]. Estimates suggest that 1 in 4 young people between the ages of 16 to 24 years have experienced at least 1 mental disorder in the preceding year [2]. Suicide, typically associated with severe distress and mental health issues, remains one of the leading causes of death among young people [3-5]. Such concerns are further exacerbated by low levels of youth help-seeking behavior for mental health issues [6,7]. Concerns about stigma and confidentiality, shame or embarrassment in discussing personal issues, financial costs, and/or limited access to services are among the many barriers to accessing help in this group [8].

In addressing the burgeoning issue of youth mental health problems, researchers and clinicians have focused on childhood and adolescence as a particularly fruitful area for targeting preventative interventions [9]. Subclinical symptoms, dysfunctional cognitive styles, and problematic behaviors which increase vulnerability to later mental health problems usually develop during this period, making childhood and adolescence an ideal time to direct prevention and early intervention programs [10-12].

To date, prevention programs for young people have tended to yield inconsistent results because of a number of factors, including the application of techniques originally developed for the treatment of clinical conditions, poor methodology, difficulties in measuring change in “normal populations,” and/or lack of relevance to some subgroups within that population [13,14]. Furthermore, programs that have been successful in reducing psychopathology in adolescents have incurred high financial costs and relied on scarce resources, such as teachers’ classroom time or researchers for program delivery [15]. A paradigm shift in mental health prevention programs is therefore required to ensure more effective and widespread delivery, improved levels of overall well-being, and high acceptability by young people.

### Positive Psychology

Positive psychology is the study of well-being, engagement, and optimal functioning and differs from clinical psychology in that it conceptualizes mental health as more than just the absence of mental illness. Positive psychology fits well with the World Health Organization’s (WHO) definition of mental health: “a state of well-being in which the individual is able to realize their potential, cope with normal life stresses, and is able to work and contribute in the community” [16]. Positive psychology is best understood as a group of mental health domains that contribute to an individual’s well-being. Although researchers debate the validity and relative importance of the various domains, a number have repeatedly been found to correspond with optimal mental health, including gratitude, mindfulness, positive social relationships, meaning, flow, optimism, hope, character strengths, and a healthy lifestyle [17]. Attempts have been made to refine these domains, such as the PERMA model proposed by Seligman [17], which stipulates that happiness is made up of 5 key components: positive emotions, engagement (similar to the concept of mindfulness), positive relationships, meaning, and accomplishment.

Evidence has accrued that addressing specific skills in these domains can promote overall well-being [18,19]. For example, interventions that increase hope have been shown to predict lower illicit substance use; lower levels of depression, anxiety, and hostility; less behavioral problems; and higher academic performance in adolescents [20,21]. Research has also linked increases in gratitude—the state of positive reflection and appreciation—to improved positive affect, life satisfaction, improved social relationships, as well as lower suicidal ideation and suicide attempts [22,23]. Longitudinal research has found that, for students faced with the challenges of entering university, higher optimism was predictive of higher well-being, better physical health, better adjustment, fewer symptoms of depression and stress, better social supports, higher levels of academic achievement, and lower dropout rates [24-27]. Optimism in adolescence has also been found to be the best predictor of life satisfaction in adulthood [28]. A meta-analysis that reviewed 51 positive psychology interventions across a spectrum of domains, found that positive psychology programs significantly increased well-being (mean *r*=.29) [29] and led to significant reductions in depressive symptoms (mean *r*=.31). These findings suggest that positive psychology may lend itself to early intervention programs targeting “normal” populations of young people.

### Online Delivery

Both eHealth and online programs provide a mode of delivery which is acceptable to youth and are financially sustainable [7,30,31]. Furthermore, users can maintain anonymity and confidentiality, determine their own rate of progression through a program, and use an online interactive format that is appealing and engaging [7]. Most young people in Australia have access to online facilities [32] as computers and Internet access are available in most schools and libraries. Other information communication technology, such as smartphones and tablets, are also becoming increasingly affordable [7]. Young people typically use online resources for dealing with distress, with a report by Mission Australia [33] quantifying that 1 in 5 young Australians (ages 11-24 years) ranked the Internet as an important source of information and support for sensitive personal issues. Similarly, ReachOut, a national youth mental health website, found that online avenues assist those who might not seek help in more traditional forms. Based on the ReachOut National Survey [34], up to 75% of young people experienced high to very high levels of psychological distress at the time of visiting the website and almost two-thirds of the sample had never accessed face-to-face mental health services.

From the perspective of service delivery, using an online format for a positive psychology program allows wider dissemination, reduces costs that would be associated with clinicians, and allows greater treatment fidelity [31,35]. Additionally, user progress can be easily monitored and the data collection process can be automated. At present, only online programs targeting single positive psychology domains have been evaluated as isolated exercises and there are no online multicomponent positive psychology programs for young people.

### The Current Study

**Figure 1:**
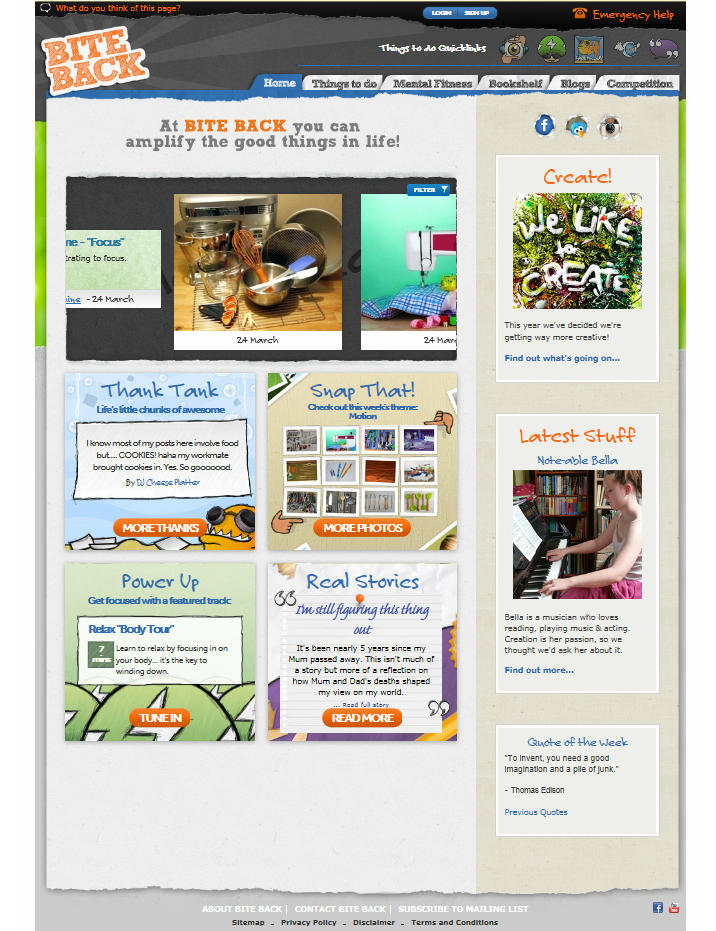
Screenshot of the Bite Back website.

This study aims to investigate the feasibility of implementing an online multicomponent positive psychology program, Bite Back (see Figure 1) developed by the Black Dog Institute, as a well-being program for young people [36].

The specific aims of this study were:

1. To examine the feasibility of an online positive psychology program for young people to improve the well-being and address mental health problems of Australian youth;
2. To investigate rates of adherence and attrition among young people who use this online positive psychology program; and
3. To investigate the acceptability/appeal of this program with young people.

## Methods

### Participants/Recruitment

Participants were recruited through schools and youth organizations across Australia. Promotional information packs advertising the How Do You View the World Study were disseminated via mail and email and included a letter to the organization, principal, and/or school counselor that detailed the rationale, requirements, and participation incentives of the study, and a series of flyers promoting the study to young people. Organizations were asked to distribute the flyers or directly promote the study to young people (aged 12-18 years) in any manner they deemed appropriate (eg, notices in newsletters, on websites, and announcements). Recruitment was conducted from November 2011 to June 2012 when it was ceased because of funding constraints. This study was approved by the University of New South Wales Human Research Ethics Advisory Panel.

Inclusion criteria were: (1) 12-18 years of age, (2) currently living in Australia, (3) having a valid email address, (4) having access to a computer with an Internet connection, and (5) providing a signed parental consent form to researchers if under 16 years of age. No exclusion criteria were applied. Researchers had no face-to-face contact with any of the participants in this study. In cases in which duplicate email addresses or participant names were found during the sign-up stage, the first application was retained and duplicates discarded.

### Study Design

#### Overview

A 2-group pretest/posttest design was used with 2 independent variables: time (baseline and postprogram) and group (Bite Back and control conditions). Dependent variables were psychological symptoms (depression, anxiety, and stress) as well as a measure of well-being. Additionally, at postintervention, usage data and qualitative feedback about the websites were collected.

#### Positive Psychology (Bite Back) Condition

Bite Back is an online positive psychology website for adolescents and uses a combination of interactive exercises and information across 9 positive psychology domains: gratitude, optimism, flow, meaning, hope, mindfulness, character strengths, healthy lifestyle, and positive relationships. Furthermore, the website provides information about the benefits of increasing well-being, methods to develop skills in each of the positive psychology domains, provides links to other relevant resources, and allows for comments and online discussions. The website is aimed at adolescents aged 13 to 17 years and is premoderated with each comment and upload being monitored and approved before becoming available for public viewing.

#### The Control Condition

**Figure 2:**
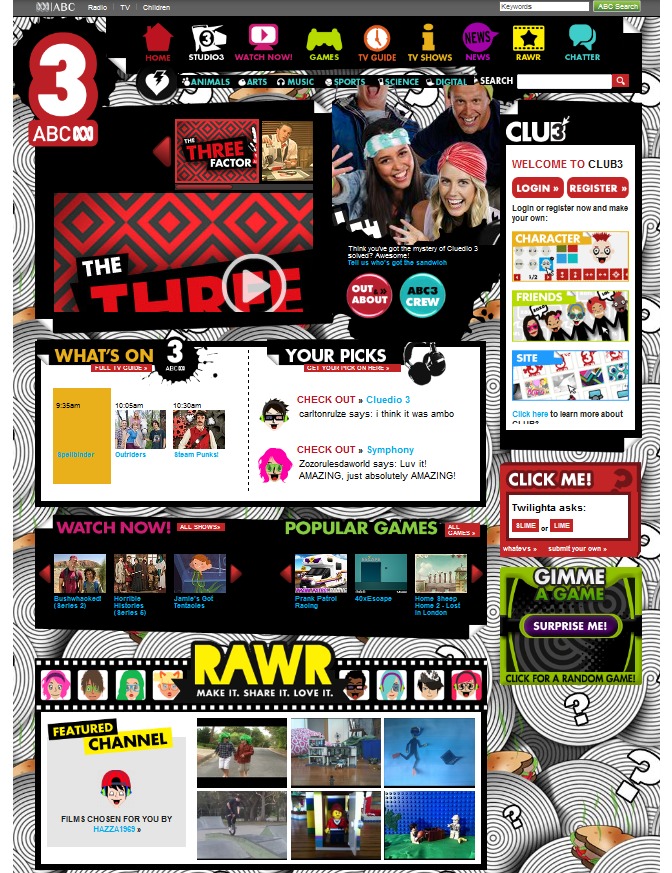
Screenshot of the ABC3 website.

**Figure 3:**
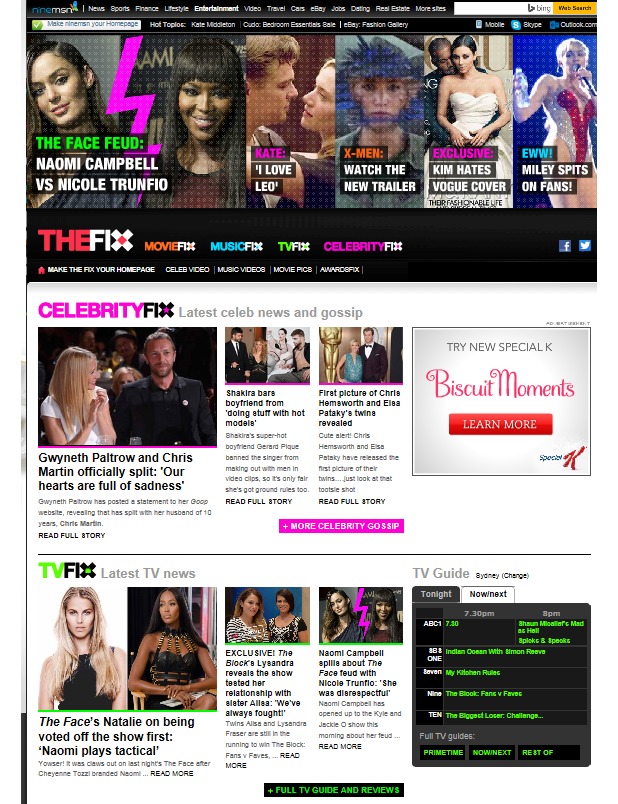
Screenshot of The Fix website.

The two control condition websites that were chosen, Australian Broadcasting Corporation digital channel website, ABC3 (see Figure 2), and Nine MSN’s entertainment website, The Fix (see Figure 3), included features that would engage young people and were similar to the Bite Back website (ie, games and/or activities). ABC3 introduces young viewers to news, comedy, drama, music, sports, and nature [37]. The Fix engages youth in popular media news, music, and videos [38]. Similar to Bite Back, both control websites are multicomponent, self-guided, youth-oriented, and Australian-based, and each has the option of contributing personal pieces of work, opinions, and stories to the website. Neither of the control websites delivers positive psychology or information about well-being.

### Study Procedures

The study was advertised as the “How Do You View the World Study: an investigation into how websites impact on the way young people think, react, and interact with the world.” It was important to conceal the clinical focus of this study and participants’ allocated condition for 2 reasons: (1) to ensure that control participants did not use the Bite Back website, and (2) to minimize any expectancy effects. Participants were also offered an AU $20 voucher for their participation in this study.

Interested young people provided their email address, name, date of birth, sex, postcode, and a parental contact email if they were younger than 16 years of age. If eligible, participants were emailed a link to the battery of baseline questionnaires. Participants who completed the baseline questionnaires were randomly allocated to one of two conditions through a block randomization method. An independent researcher not associated with this study used a random number generator in Excel to allocate blocks of 10 participants to one of two conditions:

1. Bite Back Condition: participants were instructed to create a log-in and to use Bite Back over 6 consecutive weeks.
2. Control Condition: participants were instructed to select and use either of 2 youth websites over 6 consecutive weeks, ABC3 or The Fix.

Following baseline assessment, an email was sent to participants that included a link to their allocated website and instructions on how to use it however and whenever they wanted over the next 6 weeks, but “for at least an hour a week.” Participants could access their allocated website from any Internet-enabled device and from any location. Given that all websites were open-access, it was not necessary to use the link to access the website. Both Bite Back and control participants received reminder emails once a week to encourage ongoing use and engagement with the websites. Six weeks from their date of commencement, participants were emailed the postintervention questionnaire battery and told that they no longer needed to access the website each week. Those who completed these questionnaires were emailed an AU $20 voucher from a digital media outlet.

### Measures

#### Overview

Participants completed online questionnaires before and after the 6-week intervention period. Demographic information was collected at baseline to assess eligibility and participant characteristics.

#### Adherence

Participant intervention adherence was measured by duration of exposure to the program during the course of the trial [39]. Participant’s adherence was assessed by the question “How much time did you spend actually using the website each week?” In answering this question, participants had to nominate 1 of the following categories: 0-10 minutes per week, 10-20 minutes per week, 20-30 minutes per week, 30-40 minutes per week, 40-50 minutes per week, 50-60 minutes per week, or more than 1 hour per week. Those who reported usage duration of less than 1 hour per week for their allocated website were provided a supplementary open-ended question: “If you didn’t use the website for an hour a week, was there a reason why?”

Self-reported frequency of site visits per week was also included as a measure of adherence. Participants reported their frequency of site visits by responding to the question “How many times a week did you use the site, on average?” Response options ranged from zero to more than 5 times per week.

#### Acceptability of Exercises

To determine the appeal and usability of the positive psychology program, Bite Back, participants were required to respond on a Likert scale (from 1 to 7 where 1=strongly agree and 7=strongly disagree) to 3 statements about the website: (1) “The website was fun,” (2) “The activities are interesting,” and (3) “The website was easy to use.” Intention to return to the Bite Back website after the completion of the study was also assessed.

#### Efficacy of the Program

Efficacy of the program was assessed by self-report questionnaires at baseline and at 6-weeks via:

1. The Depression, Anxiety, and Stress Scale—Short form (DASS-21) [40] comprises 3 symptom-based subscales. Each subscale has 7 items which participants respond to on a 4-point Likert scale (0=not at all to 3=most of the time). Summed scores for each scale range from 0-42 following conversion of scores to match the DASS-42; more severe symptoms are indicated by higher scores. The DASS-21 has been used with adolescent samples and is reported to have a Cronbach alpha of .87 for depression, .79 for anxiety, and .83 for the stress subscales [41]. The DASS has also been demonstrated to correlate closely with the *Diagnostic and Statistical Manual of Mental Disorders* (*DSM*) diagnoses of panic disorder, generalized anxiety disorder, social phobia, simple phobia, and major depressive disorder [42,43]. The DASS-21 was modified slightly to aid comprehension of the wording for adolescents. These changes were approved by the authors and the original meaning of the items remained unchanged.
2. The Short Warwick-Edinburgh Mental Well-Being Scale (SWEMWBS) [44] is a 7-item measure that assesses participants’ experiences of subjective positive mental health (well-being) over the past 2 weeks on a 5-point scale (1=none of the time to 5=all the time). Psychometric data of the measure on the original WEMWBS for adolescents has indicated satisfactory to high internal consistency (*r*=.87) and the short version has acceptable test-retest reliability (*r*=.66, 95% CI 0.59-0.72) [44,45]. In an adolescent population, the SWEMWBS has been demonstrated to be negatively correlated with the Strength’s and Difficulties Questionnaire, a measure of adolescent psychopathology (*r*=–0.44, 95% CI –0.49 to –0.40), negatively correlated with the 12-item General Health Questionnaire (*r*=–.45, 95% CI –0.49 to –0.40), and positively correlated with both the WHO-Five Well-being Index (*r*=.57, 95% CI 0.53-0.61) [44].

## Results

### Attrition and Sample Characteristics

**Figure 4:**
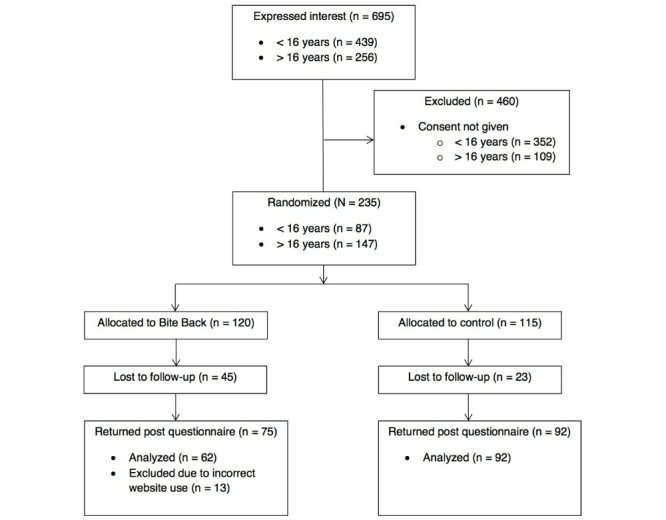
CONSORT diagram of participants.

Of the 695 participants who expressed interest in the study, 235 met the inclusion criteria. The 235 participants who met the inclusion criteria were allocated to either the Bite Back or control condition and completed baseline questionnaires. After the 6-week trial period, 167 participants remained in the study and completed the follow-up questionnaires. A further 13 participants in the intervention condition were deemed to be noncompliant because they reported using the incorrect website (see Figure 4) as per protocol [46,47]. Noncompliant participants and noncompleters were excluded from the analysis. The researchers who conducted the analyses were not blinded to the allocated condition of participants. Figure 4 displays the consolidated standards of reporting trials (CONSORT) diagram for progression of participants at each stage of the evaluation.

There were no significant age or gender differences between completers and dropouts. Furthermore, completers and dropouts were equivalent on baseline scores for DASS-21 depression (mean 9.0, SD 8.5 vs mean 10.3, SD 9.0), anxiety (mean 6.4, SD 7.0 vs mean 7.5, SD 7.7), and the SWEMWBS (mean 24.8, SD 5.0 vs mean 23.8, SD 5.1). A significant difference (*F*_1,211_=4.22, *P*=.04), between completers and dropouts was found on baseline stress scores (mean 10.7, SD 7.9 vs mean 13.2, SD 8.7).

The final sample of 154 participants was comprised of 104 (67.5%) females with a mean age of 15.4 (SD 1.7) years. At baseline, both Bite Back and control condition participants were equivalent in mean age, gender distribution, and mean scores on the DASS-21 depression, anxiety, and stress subscales and on the SWEMWBS (see Table 1).

Participants were divided into 2 groups based on the frequency of visits to their allocated website: low frequency (less than 3 site visits per week) and high frequency (3 or more site visits per week). There were no significant baseline differences in DASS-21 subscales or SWEMWBS scores across conditions for frequency of site visits. Participants were also divided into 2 groups based on the amount of time they spent on their assigned website: low (30 minutes or less per week) and high (30 minutes or more per week). There were no significant baseline differences in DASS-21 subscales or SWEMWBS scores across conditions for duration of site visits.

**Table 1:** Characteristics of the final sample (those who completed baseline and postintervention measures) for each condition.

Characteristic		Bite Back, n=62	Control, n=92	Total, n=154
Age (years), mean (SD)		15.5 (1.6)	15.3 (1.7)	15.4 (1.7)
Female, n (%)		40 (64.5)	64 (69.6)	104 (67.5)
**Baseline DASS depression, n**		59	80	139
	Mean (SD)	9.4 (8.3)	8.6 (8.7)	8.9 (8.5)
**Baseline DASS anxiety, n**		53	86	139
	Mean (SD)	7.2 (6.9)	6.0 (7.3)	6.4 (7.2)
**Baseline DASS stress, n**		53	86	139
	Mean (SD)	11.7 (7.3)	9.9 (8.7)	10.6 (7.9)
**Baseline SWEMWBS, n**		58	87	145
	Mean (SD)	24.1 (5.2)	25.7 (5.4)	24.8 (5.0)

### Adherence

There were no significant differences between the reported duration spent using the websites each week between those allocated to the intervention and control conditions. In the intervention condition, 14 of the 61 participants (23%) reported using the website for between 50-60 minutes, 6 (10%) participants reported usage of between 40-50 minutes per week, and 37 (61%) participants reported usage less than 40 minutes per week. In the control condition, 26 of the 90 participants (29%) reported using their website for between 50-60 minutes, 14 (16%) participants reported usage between 40-50 minutes per week, and 46 (51%) of control participants reported usage at a rate less than two-thirds of the recommended duration.

Participants in the Bite Back condition who reported using the website for less than an hour a week were asked to provide a reason for their underusage. In all, 36 participants responded to the question “If you didn’t use the website for an hour a week, was there a reason why?” Responses were analyzed to extract key themes and coded to capture primary thematic components. Four main themes emerged: time constraints, technical issues, relevance, and website content.

Of the 36 participants who responded, 21 (58%) cited that the reason for their underusage was time constraints. This related to demands on time such as schoolwork, extracurricular activities, hobbies, family commitments, and going away on holidays. Technical issues accounted for 5 participants’ (14%) underusage, predominantly issues with Internet access:

It was hard to complete certain tasks and areas of the website as my data had run out on my Internet.

participant 444, male, age 16 years

Four participants (11%) reported that Bite Back did not seem relevant for them:

Sorry I lost interest, because although I thought it was a great site I didn’t think it was relevant for me.

participant 352, female, age 17 years

The site’s design was not appealing to me, it looked directed at 10-13 year olds.

participant 115, female, age 17 year

Furthermore, 6 participants (17%) stated that the content of the website was not sufficient to sustain their interest for an hour a week:

Honestly the first week, the website was pretty new to you, so to get around you had to learn your way, therefore it was pretty cool yeah? But then afterwards it was all too repetitive and much too the same, things I knew already there.

participant 161, female, age 16 years

The website was very similar each time I visited it and thus lost the initial flair it once had.

participant 609, male, age 14 years

### Acceptability of Exercises

Participants in the Bite Back condition were also asked to provide feedback on the appeal and usability of the positive psychology program. Of the 62 participants who used the program for 6 weeks, 49 reported that Bite Back was fun (79% rated mildly agree to strongly agree), with 52 agreeing that the activities were interesting (84% rated mildly agree to strongly agree), and 56 reporting that the site was easy to use (90% rated mildly agree to strongly agree).

Of the 62 participants in the Bite Back condition, 35 participants (57%) responded yes and 19 participants (31%) responded maybe. Only 7 participants (11%) responded that they would not revisit Bite Back in the future (1 participant gave no response).

### Efficacy of the Program

To analyze the efficacy of the program, a series of 2-tailed Wilcoxon signed rank tests were conducted to measure differences in psychopathology and well-being scores before and after the intervention. Nonparametric tests were employed because of violations of the normality assumption for almost all group cells (condition; condition × frequency of site visits; condition × length of site visits). Only the postintervention scores on the SWEMWBS did not demonstrate violations of normality. All violations were because of skew in the data that would be expected in a nonclinical population (ie, low scores on the DASS-21 and high scores on the SWEMWBS).

Participants in the Bite Back condition returned significantly lower DASS-21 depression (*z*=–2.44, *P*=.02, *r*=–.22) and stress scores (*z*=–2.14, *P*=.03, *r*=–.21) at 6 weeks postintervention. In addition, they returned significantly higher scores on the SWEMWBS (*z*=2.07, *P*=.04, *r*=.19). No significant differences in DASS-21 subscales or SWEMWBS scores were found in the control condition from preassessment to postassessment (see Table 2).

Samples for each condition were dichotomized (high and low) based on frequency of site visits and time spent on the site each week to derive approximately equal numbers in each subgroup. Two further Wilcoxon signed rank tests were conducted to determine if frequency of use or length of time of use affected postintervention levels of symptoms and well-being.

Participants who visited their corresponding site 2 times per week or less were classified as having low frequency of use, whereas those visiting their allocated website 3 or more times per week were classified as high frequency of use. Participants in the Bite Back condition who visited the site with high frequency reported significant reductions in depression (*z*=–2.39, *P*=.02, *r*=–.34) and anxiety (*z*=–1.98, *P*=.05, *r*=–.29) scores, as well as significant increases in well-being scores (*z*=2.28, *P*=.02, *r*=.34) from preintervention to postintervention. Participants in this group also reported a reduction in stress symptoms that approached significance (*z*=–1.80, *P*=.07, *r*=–.28) (see Table 3). No such differences were found in the control condition.

Participants who visited their allocated website for 30 minutes or less per week were classified as low duration of use, whereas those who used their website for 30 minutes or more per week were classified as high duration of use. Participants in the Bite Back group who visited the site for 30 minutes or more per week (ie, in the high duration group) reported significant reductions in depression (*z*=–2.57, *P*=.01, *r*=–.33) and stress (*z*=–2.74, *P*=.006, *r*=–.37) scores at postintervention. This group also reported a trend for lower anxiety (*z*=–1.92, *P*=.06, *r*=–.26) and increased well-being scores (*z*=1.89, *P*=.06, *r*=.24), but these differences failed to reach significance (see Table 4). A significant increase in anxiety scores at postintervention (*z*=2.95, *P*=.003, *r*=.35) was found in control condition participants who reported low levels of time spent on their assigned websites. A significant increase in well-being scores at postintervention, (*z*=2.00, *P*=.05, *r*=.20) was found in control condition participants who reported high levels of time spent on their assigned websites.

**Table 2:** Preprogram and postprogram DASS-21 and SWEMWBS scores for each condition.

Variable and condition		Baseline		Post		*P*
		Mean (SD)	n	Mean (SD)	n	
**Depression**						
	Bite Back	9.39 (8.26)	59	6.68 (7.72)	59	.02
	Control	8.60 (8.73)	80	8.28 (8.48)	80	.83
**Anxiety**						
	Bite Back	7.17 (6.91)	53	6.38 (7.26)	53	.27
	Control	5.98 (7.31)	86	6.86 (8.61)	86	.09
**Stress**						
	Bite Back	11.66 (7.35)	53	10.08 (8.10)	53	.03
	Control	9.88 (8.17)	86	9.35 (8.69)	86	.42
**Well-being**						
	Bite Back	24.12 (5.16)	58	25.78 (5.35)	58	.04
	Control	25.17 (4.87)	87	25.75 (5.43)	87	.23

**Table 3:** Preprogram and postprogram DASS-21 and SWEMWBS scores by frequency of site visits for each condition.

Variable, condition, and frequency			Baseline		Post		*P*
			Mean (SD)	n	Mean (SD)	n	
**Depression**							
	**Bite Back**						
		Low	7.82 (6.31)	33	7.09 (7.65)	33	.30
		High	11.84 (9.91)	25	6.40 (7.98)	25	.02
	**Control**						
		Low	8.24 (9.36)	34	7.18 (7.96)	34	.69
		High	8.36 (7.64)	45	8.80 (8.72)	45	.73
**Anxiety**							
	**Bite Back**						
		Low	6.69 (6.57)	29	7.17 (8.53)	29	.71
		High	8.09 (7.37)	23	5.65 (5.38)	23	.05
	**Control**						
		Low	4.67 (6.40)	36	5.33 (6.56)	36	.21
		High	6.86 (7.91)	49	7.88 (9.84)	49	.24
**Stress**							
	**Bite Back**						
		Low	12.654 (7.82)	31	12.00 (8.75)	31	.18
		High	10.76 (6.28)	21	7.62 (6.38)	21	.07
	**Control**						
		Low	9.46 (9.28)	37	8.81 (8.27)	37	.81
		High	10.13 (7.36)	48	9.58 (9.07)	48	.36
**Well-being**							
	**Bite Back**						
		Low	24.65 (4.10)	34	25.24 (5.09)	34	.50
		High	23.04 (6.33)	23	26.30 (5.74)	23	.02
	**Control**						
		Low	24.81 (5.43)	37	25.59 (5.07)	37	.32
		High	25.44 (4.45)	50	25.86 (5.73)	50	.45

**Table 4:** Preprogram and postprogram DASS-21 and SWEMWBS scores by duration of use for each condition.

Variable, condition, and length of visit			Baseline		Post		*P*
			Mean (SD)	n	Mean (SD)	n	
**Depression**							
	**Bite Back**						
		Low	8.22 (7.77)	27	7.78 (9.37)	27	.52
		High	10.71 (8.57)	31	5.94 (5.99)	31	.01
	**Control**						
		Low	9.26 (8.42)	35	9.49 (9.42)	35	.69
		High	8.18 (9.11)	44	7.18 (7.66)	44	.34
**Anxiety**							
	**Bite Back**						
		Low	7.04 (7.77)	25	7.68 (9.07)	25	.72
		High	7.56 (6.14)	27	5.41 (5.05)	27	.06
	**Control**						
		Low	7.11 (8.40)	36	9.22 (10.74)	36	.003
		High	5.22 (6.44)	49	4.90 (6.06)	49	.53
**Stress**							
	**Bite Back**						
		Low	10.83 (7.62)	24	11.67 (9.99)	24	.95
		High	12.79 (6.89)	28	9.00 (5.98)	28	.006
	**Control**						
		Low	11.29 (8.68)	34	11.35 (9.51)	34	.75
		High	9.04 (7.92)	50	7.84 (7.80)	50	.09
**Well-being**							
	**Bite Back**						
		Low	24.40 (5.38)	25	25.12 (5.75)	25	.36
		High	23.69 (4.99)	32	26.09 (5.04)	32	.06
	**Control**						
		Low	24.14 (5.37)	36	24.19 (5.65)	36	.87
		High	25.88 (4.47)	49	26.88 (5.13)	49	.05

## Discussion

### Principal Findings

This study examined the feasibility and challenges of an online, open-access, positive psychology program (Bite Back) to increase well-being and reduce mental health symptoms in young people. Despite difficulties maintaining high levels of adherence and low levels of attrition, positive qualitative feedback from participants indicated that adolescents enjoyed Bite Back and found it interesting and easy to use. In addition, significant improvements in symptoms of depression, stress, and well-being scores were observed for the Bite Back condition.

Further investigation also suggested that those who visited the Bite Back website more frequently during the intervention period and for greater amounts of time each week gained the most benefit. Participants in at least one of these high-adherence groups reported lower depression, anxiety, and stress scores, and higher well-being scores at postintervention. The beneficial outcomes at 6 weeks are particularly noteworthy given the short-term and entirely self-directed nature of this intervention.

However, the most beneficial outcomes were present among participants who visited the website for 30 minutes or more during each week of the intervention period or 3 or more times during each week of the intervention. No significant changes were found among Bite Back users who did not meet these levels of adherence indicating that the program requires a certain level of usage for a positive effect. Overall, these positive findings suggest that further research into Bite Back’s effectiveness is warranted, particularly if adolescents’ engagement with the site can be improved.

Retention rates for the Bite Back condition were moderate. A total of 167 of 235 participants who completed the battery of questionnaires at baseline also completed the battery after the 6-week trial period: retention rates across time periods were 71% for the total sample and 58% for the Bite Back condition alone (although only 66% of the total sample and 52% of the Bite Back condition were used in the analysis because of the exclusion of the noncompliant participants). A systematic review of attrition rates from Internet-based treatment programs where therapist contact was minimal found an average dropout rate of 35%, with a range of 2% to 83% [46]. Although that review was based on psychological treatment programs for adults, it suggests that the current trial returned an acceptable attrition rate in the context of eHealth programs. One unexpected finding was that the large discrepancy between the attrition and noncompliance rates for the Bite Back condition compared to the control condition. The only factor examined that differed significantly between completers and dropouts were baseline stress scores, with the attrition group reporting significantly higher stress at the start of the program. Although the baseline stress scores did not differ significantly between the Bite Back and control conditions, the Bite Back condition reported higher baseline stress scores. It is possible to speculate, therefore, that the stress they were experiencing interfered with their ability to stay engaged with the program.

Approximately half the participants’ self-reported usage was below the prescribed 1 hour per week over the 6-week trial period. However, these rates of usage were equivalent across both the intervention and control websites. Therefore, it is likely that these low levels of adherence are reflective of the more general methodological challenges associated with open-access trials [39], as opposed to a particular failing of Bite Back to sustain participants’ attention.

This interpretation is further supported by qualitative reports from Bite Back participants. Primary reasons cited for underusage were time constraints (related to school, extracurricular, and family commitments) and technical issues, such as problems in accessing the Internet. In addition, acceptability ratings of Bite Back were favorable: 79% agreed the program was fun, 84% agreed that it was interesting, and 90% agreed that it was easy to use. Most users stated an intention to return to Bite Back after the completion of the study. Users who were disappointed (11%) with Bite Back cited content relevance and lack of age appropriateness. Thus, it seems evident that although logistical issues were primarily related to low adherence rates, website content also affected users’ continued engagement.

Bite Back users who reported using the website for 30 minutes or less per week or fewer than 3 times per week were predominantly older than 16 years of age and stated that the content had little relevance or was not sufficiently interesting to sustain their attention. These findings suggest that Bite Back may have more appeal for those 16 years of age or younger. Developmentally, adolescence is a time of rapid change, particularly emotional and intellectual. As such, there is considerable heterogeneity across the age 12 to 18 years group that makes it difficult to pitch activities and conceptual explanations that are universally appropriate. Further data are needed to assess whether age is a specific factor for content relevance, which age groups most strongly identified with the site, and which positive psychology domains or site activities were effective at maintaining engagement for older adolescents.

### Limitations of the Study

Despite the encouraging results from this pilot study, methodological limitations preclude us from drawing definitive conclusions. Our study may have been affected by measurement sensitivity (ie, the psychopathology measure selected was designed for a clinical population and so floor effects could have impacted on our results). This problem may have been exacerbated by the small sample sizes in each condition making it less likely to obtain significant differences.

Furthermore, our usage data heavily relied on participants’ self-report which could have been affected by memory and reporting biases [48]. In addition, adolescent samples may be likely to underreport mental health symptoms [48,49], although the anonymous nature of this study may have lessened this likelihood. Utilizing corroborative evidence from teachers and parents may have improved the accuracy of our measurements of change in symptom alleviation and well-being and should be considered for further studies in this area.

The study gathered limited information about participants’ use of Bite Back, such as content accessed, uploads to interactive activities, and time spent on the various activities. Because the website was comprised of multiple components, including videos, psychoeducational information, interactive exercises, and community noticeboards, it is difficult to ascertain which parts of the website were instrumental to the changes observed. Information on usage patterns would have provided interesting insights of how young people navigate the website and a better understanding of the differential impact of its various components. Further research into this area would shed light on the way in which young people relate to specific online positive psychology interventions.

The age distribution of our population was also an important factor in considering the results of this study. Although a larger percentage of those younger than 16 year expressed interest in participating, fewer of the participants younger than 16 years progressed through to actually participate in the study because of the need for them to obtain parental permission. Given that our qualitative responses suggested that Bite Back may be more acceptable to the younger age group, this barrier to participation by younger users may have excluded an important segment of our target population in this trial.

Although Bite Back was developed as a preventative program for youth, this feasibility study was primarily concerned with obtaining feedback on users’ enjoyment and willingness to engage in the site’s activities. As such, the preliminary pilot data from this study is insufficient to demonstrate preventative effects. Further studies utilizing a longer-term follow-up are necessary to examine this important aspect of preventative health in youth.

### Conclusions

This study explored the feasibility of an online, open-access, multicomponent, positive psychology program for adolescents. The findings from this study demonstrate that an online positive psychology program (Bite Back) has the potential to reduce symptoms of psychopathology and promote mental well-being. However, issues of adherence and age appropriateness need to be addressed for optimal outcomes. Nevertheless, the positive findings from this study suggest that online positive psychology programs may have a major impact on the emerging field of adolescent-focused eHealth for improving well-being and resilience.

## Multimedia Appendix 1

CONSORT-EHEALTH checklist V1.6.2 [50].

## Abbreviations

ABC: Australian Broadcasting Corporation
CONSORT: Consolidated Standards of Reporting Trials
DASS-21: Depression, Anxiety, Stress Scale—short form
SWEMWBS: Short Warwick-Edinburgh Mental Well-Being Scale
