# Supplementary material for: Feasibility and Effectiveness of a Web-Based Positive Psychology Program for Youth Mental Health: Randomized Controlled Trial
Source: J Med Internet Res. 2014 Jun 4;16(6):e140. doi: 10.2196/jmir.3176 (PMC4071231; doi:10.2196/jmir.3176)
Supplement: Supplementary file 1 [file jmir_v16i6e140_app1.pdf]

CONSORT-EHEALTH Checklist V1.6.2 Report

(based on CONSORT-EHEALTH V1.6), available at [http://tinyurl.com/consort-ehealth-v1-6].

3176

Date completed

3/11/2014 5:11:21

by

Vijaya Manicavasagar

Positive Psychology for Young People: Feasibility of Online Delivery

TITLE

1a-i) Identify the mode of delivery in the title

Online delivery is identified in the study title.

1a-ii) Non-web-based components or important co-interventions in title

1a-iii) Primary condition or target group in the title

The title specifies the target group as 'young people'.

ABSTRACT

1b-i) Key features/functionalities/components of the intervention and comparator in the METHODS section of the ABSTRACT

A brief overview of the intervention and control conditions are included in the abstract:

"...positive psychology (PP) holds promise for both prevention and intervention, building resilience and improving the wellbeing of young people.

Objectives:
This study explores the feasibility of the online delivery of a youth PP program, ‘Bite Back’, to improve the wellbeing and mental health outcomes of Australian youth. Further aims were to examine rates of adherence and attrition, and to investigate the acceptability of this program for young people.

Methods:
Participants (n = 235) aged 12 -18 years were recruited throughout Australia and randomly assigned to either of two conditions: Bite Back (n = 120) or control websites (n = 115)..."

1b-ii) Level of human involvement in the METHODS section of the ABSTRACT

1b-iii) Open vs. closed, web-based (self-assessment) vs. face-to-face assessments in the METHODS section of the ABSTRACT

1b-iv) RESULTS section in abstract must contain use data

1b-v) CONCLUSIONS/DISCUSSION in abstract for negative trials

INTRODUCTION

2a-i) Problem and the type of system/solution

"Addressing mental health problems in young people is a major public health concern [1] with estimates suggesting that one in four young people between the ages of 16 to 24 years have experienced at least one mental disorder in the preceding year [2]. Furthermore, suicide, typically associated with severe distress and mental health issues, remains one of the leading causes of death amongst young people [3-5]. Such concerns are further exacerbated by low levels of youth help-seeking behaviour for mental health issues [6, 7]. Concerns about stigma and confidentiality, shame or embarrassment in discussing personal issues, financial costs and/or limited access to services are amongst the many barriers to accessing help in this group [8] "

"A paradigm shift in mental health prevention programs is therefore required to ensure more effective and widespread delivery, improved levels of overall wellbeing and high acceptability by young people."

"From the perspective of service delivery, using an online format for a PP program allows wider dissemination, reduces costs that would be associated with clinicians and allows greater treatment fidelity [31, 35]. Additionally user progress can be easily monitored and data collection process can be automated. At present, only online programs targeting single PP domains have been evaluated as isolated exercises and there are no online, multi-component PP programs for young people."

2a-ii) Scientific background, rationale: What is known about the (type of) system

"Evidence has accrued that addressing specific skills in these domains can promote overall wellbeing [18, 19]. For example, interventions that increase ‘Hope’ have been shown to predict lower illicit substance use, lower levels of depression, anxiety and hostility, less behavioural problems and higher academic performance in adolescents [20, 21]. Research has also linked increases in Gratitude – the state of positive reflection and appreciation – to improved positive affect, life satisfaction, improved social relationships as well as lower suicidal ideation and suicide attempts [22,23]. Longitudinal research has found that, for students faced with the challenges of entering university, higher Optimism was predictive of higher wellbeing, better physical health, better adjustment, fewer symptoms of depression and stress, better social supports, higher levels of academic achievement, and lower drop-out rates [24-27]. Optimism in adolescence has also been found to be the best predictor of life satisfaction in adulthood [28]. A meta-analysis that reviewed 51 PP interventions across the spectrum of domains, found that on average PP programs significantly increased wellbeing (mean r =.29) [29] and led to significant reductions in depressive symptoms (mean r = .31). These findings suggest that PP may lend itself well to early intervention programs targeting ‘normal’ populations of young people"

"E-health or online programs provide a mode of delivery which is acceptable to youth and are financially sustainable [7, 30, 31]. Furthermore, users can maintain anonymity and confidentiality, determine their own rate of progression through a program, and use an online interactive format that is appealing and engaging [7]. Most young people in Australia have access to online facilities [32] as computers and internet access are available in most schools and libraries. Other information communication technology (ICT), such as smart phones and tablets, are also becoming increasingly affordable [7]. Young people typically use online resources for dealing with distress, with a report by Mission Australia [33] quantifying that one in five young Australians (ages 11 – 24 years) ranked the internet as an important source of information and support for sensitive personal issues. Similarly ReachOut, a national youth mental health website, found that online avenues assist those who might not seek help in more traditional forms. Based on the ReachOut National Survey [34], up to 75% of young people experienced ‘high’ to ‘very high’ levels of psychological distress at the time of visiting the website and almost two-thirds of the sample had never accessed face-to-face mental health services."

METHODS

3a) CONSORT: Description of trial design (such as parallel, factorial) including allocation ratio
"This study aims to investigate the feasibility of implementing an online multi-component PP program, Bite Back, developed by the Black Dog Institute, as a wellbeing program for young people [36].
The specific aims of this study were:
1) To examine the feasibility of an online PP program for young people to improve the wellbeing and address mental health problems of Australian youth
2) To investigate rates of adherence and attrition amongst young people who use this online PP program; and
3) To investigate the acceptability/appeal of this program with young people"
3b) CONSORT: Important changes to methods after trial commencement (such as eligibility criteria), with reasons
There were no changes to methods after trial commencement.
3b-i) Bug fixes, Downtimes, Content Changes

4a) CONSORT: Eligibility criteria for participants
"Participants were recruited through schools and youth organisations across Australia. Promotional information packs advertising the 'How Do You View The World Study' were disseminated via mail and email and included a letter to the organisation/principal/school counsellor that detailed the rationale, requirements and participation incentives of the study, and a series of flyers promoting the study to young people. Organisations were asked to distribute the flyers or directly promote the study to young people (12- 18 years old) in any manner they deemed appropriate (e.g. notices in newsletters, on websites, and announcements). This study was approved by the UNSW Human Research Ethics Advisory panel.
Inclusion criteria were: (1) 12 -18 years of age, (2) currently living in Australia, (3) having a valid email address, (4) having access to a computer with an internet connection, and (5) providing a signed parental consent form to researchers if under 16 years of age. Researchers had no face-to-face contact with any of the participants in this study."
4a-i) Computer / Internet literacy

4a-ii) Open vs. closed, web-based vs. face-to-face assessments:
"Participants were recruited through schools and youth organisations across Australia. Promotional information packs advertising the 'How Do You View The World Study' were disseminated via mail and email and included a letter to the organisation/principal/school counsellor that detailed the rationale, requirements and participation incentives of the study, and a series of flyers promoting the study to young people. Organisations were asked to distribute the flyers or directly promote the study to young people (12- 18 years old) in any manner they deemed appropriate (e.g. notices in newsletters, on websites, and announcements)."

"Inclusion criteria were: (1) 12 -18 years of age, (2) currently living in Australia, (3) having a valid email address, (4) having access to a computer with an internet connection, and (5) providing a signed parental consent form to researchers if under 16 years of age. Researchers had no face-to-face contact with any of the participants in this study."
4a-iii) Information giving during recruitment

4b) CONSORT: Settings and locations where the data were collected
"Following baseline assessment, an email was sent to participants which included a link to their allocated website and instructions on how to use it "however and whenever" they wanted over the next 6 weeks, but "for at least an hour a week". Both Bite Back and control participants received reminder emails once a week to encourage ongoing usage of and engagement with the websites. Six weeks from their date of commencement, participants were emailed the post-intervention questionnaire battery and told that they no longer needed to access the website each week."
4b-i) Report if outcomes were (self-)assessed through online questionnaires
"If eligible, participants were emailed a link to the battery of baseline questionnaires."

"Following baseline assessment, an email was sent to participants which included a link to their allocated website and instructions on how to use it "however and whenever" they wanted over the next 6 weeks, but "for at least an hour a week". Both Bite Back and control participants received reminder emails once a week to encourage ongoing usage of and engagement with the websites. Six weeks from their date of commencement, participants were emailed the post-intervention questionnaire battery and told that they no longer needed to access the website each week."
4b-ii) Report how institutional affiliations are displayed

5) CONSORT: Describe the interventions for each group with sufficient details to allow replication, including how and when they were actually administered
5-i) Mention names, credential, affiliations of the developers, sponsors, and owners

5-ii) Describe the history/development process

5-iii) Revisions and updating

5-iv) Quality assurance methods

5-v) Ensure replicability by publishing the source code, and/or providing screenshots/screen-capture video, and/or providing flowcharts of the algorithms used

5-vi) Digital preservation

5-vii) Access
"…an email was sent to participants which included a link to their allocated website and instructions on how to use it "however and whenever" they wanted over the next 6 weeks, but "for at least an hour a week". Both Bite Back and control participants received reminder emails once a week to encourage ongoing usage of and engagement with the websites"

"Firstly, to ensure that control participants did not use the Bite Back website, and secondly, to minimize any expectancy effects. Participants were also offered a $20 voucher for their participation in this study."

"Those who completed these questionnaires were emailed a $20 voucher from a digital media outlet."
5-viii) Mode of delivery, features/functionalities/components of the intervention and comparator, and the theoretical framework

"The PP (Bite Back) Condition: Bite Back is an online PP website for adolescents and uses a combination of interactive exercises and information across nine PP domains: Gratitude, Optimism, Flow, Meaning, Hope, Mindfulness, Character Strengths, Healthy Lifestyle and Positive Relationships. Furthermore, the website provides information about the benefits of increasing wellbeing; methods to develop skills in each of the PP domains; provides links to other relevant resources; and allows for comments and online discussions. The website is aimed at adolescents aged 13 to 17 years and is pre-moderated with each comment and upload being monitored and approved before becoming available for public viewing.

The Control Condition: The two control condition websites that were chosen, 'ABC3' and 'The Fix', included features that would engage young people and bore similarities to the Bite Back website (i.e.: games and/or activities). 'ABC3' introduces young viewers to news, comedy, drama, music, sports and nature [37]. 'The Fix' engages youth in popular media news, music and videos [38]. Similar to Bite Back, both control websites are multi-component, self-guided, youth-oriented and Australian-based and each has the option of contributing personal pieces of work, opinions and stories to the website. Neither of the control websites delivers PP or information about wellbeing."

5-ix) Describe use parameters

5-x) Clarify the level of human involvement

5-xi) Report any prompts/reminders used

"Both Bite Back and control participants received reminder emails once a week to encourage ongoing usage of and engagement with the websites."

5-xii) Describe any co-interventions (incl. training/support)

No co-interventions were used:

"Researchers had no face-to-face contact with any of the participants in this study."

"The PP (Bite Back) Condition: Bite Back is an online PP website for adolescents and uses a combination of interactive exercises and information across nine PP domains: Gratitude, Optimism, Flow, Meaning, Hope, Mindfulness, Character Strengths, Healthy Lifestyle and Positive Relationships. Furthermore, the website provides information about the benefits of increasing wellbeing; methods to develop skills in each of the PP domains; provides links to other relevant resources; and allows for comments and online discussions. The website is aimed at adolescents aged 13 to 17 and is pre-moderated with each comment and upload being monitored and approved before becoming available for public viewing."

"Following baseline assessment, an email was sent to participants which included a link to their allocated website and instructions on how to use it "however and whenever" they wanted over the next 6 weeks, but "for at least an hour a week". Both Bite Back and control participants received reminder emails once a week to encourage ongoing usage of and engagement with the websites. Six weeks from their date of commencement, participants were emailed the post-intervention questionnaire battery and told that they no longer needed to access the website each week. Those who completed these questionnaires were emailed a $20 voucher from a digital media outlet."

6a) CONSORT: Completely defined pre-specified primary and secondary outcome measures, including how and when they were assessed

As a feasibility study, this study did not have pre-specified primary and secondary outcome measures. Please note that the feasibility as a primary aim of the study is well articulated:

"The specific aims of this study were:

1) To examine the feasibility of an online PP program for young people to improve the wellbeing and address mental health problems of Australian youth
2) To investigate rates of adherence and attrition amongst young people who use this online PP program; and
3) To investigate the acceptability/appeal of this program with young people"

6a-i) Online questionnaires: describe if they were validated for online use and apply CHERRIES items to describe how the questionnaires were designed/deployed

6a-ii) Describe whether and how "use" (including intensity of use/dosage) was defined/measured/monitored

6a-iii) Describe whether, how, and when qualitative feedback from participants was obtained

6b) CONSORT: Any changes to trial outcomes after the trial commenced, with reasons

There were no changes to trial outcomes after the trial commenced.

7a) CONSORT: How sample size was determined

7a-i) Describe whether and how expected attrition was taken into account when calculating the sample size

7b) CONSORT: When applicable, explanation of any interim analyses and stopping guidelines

Not applicable.

8a) CONSORT: Method used to generate the random allocation sequence

"An independent researcher not associated with this study used a random number generator in Excel to allocate blocks of ten participants to one of two conditions..."

8b) CONSORT: Type of randomisation; details of any restriction (such as blocking and block size)

"An independent researcher not associated with this study used a random number generator in Excel to allocate blocks of ten participants to one of two conditions..."

9) CONSORT: Mechanism used to implement the random allocation sequence (such as sequentially numbered containers), describing any steps taken to conceal the sequence until interventions were assigned

"The study was advertised as the "How Do You View the World Study: an investigation into how websites impact on the way young people think, react and interact with the world". It was important to conceal the clinical focus of this study for two reasons. Firstly, to ensure that control participants did not use the Bite Back website, and secondly, to minimize any expectancy effects."

"An independent researcher not associated with this study used a random number generator in Excel to allocate blocks of ten participants to one of two conditions..."

10) CONSORT: Who generated the random allocation sequence, who enrolled participants, and who assigned participants to interventions

"An independent researcher not associated with this study used a random number generator in Excel to allocate blocks of ten participants to one of two conditions..."

11a) CONSORT: Blinding - If done, who was blinded after assignment to interventions (for example, participants, care providers, those assessing outcomes) and how
11a-i) Specify who was blinded, and who wasn't
"The study was advertised as the "How Do You View the World Study: an investigation into how websites impact on the way young people think, react and interact with the world". It was important to conceal the clinical focus of this study for two reasons. Firstly, to ensure that control participants did not use the Bite Back website, and secondly, to minimize any expectancy effects."

"An independent researcher not associated with this study used a random number generator in Excel to allocate blocks of ten participants to one of two conditions..."

11a-ii) Discuss e.g., whether participants knew which intervention was the "intervention of interest" and which one was the "comparator"Informed consent procedures (4a-ii) can create biases and certain expectations - discuss e.g., whether participants knew which intervention was the "intervention of interest" and which one was the "comparator".
11a-ii) Discuss e.g., whether participants knew which intervention was the "intervention of interest" and which one was the "comparator"

11b) CONSORT: If relevant, description of the similarity of interventions
"The PP (Bite Back) Condition: Bite Back is an online PP website for adolescents and uses a combination of interactive exercises and information across nine PP domains: Gratitude, Optimism, Flow, Meaning, Hope, Mindfulness, Character Strengths, Healthy Lifestyle and Positive Relationships. Furthermore, the website provides information about the benefits of increasing wellbeing; methods to develop skills in each of the PP domains; provides links to other relevant resources; and allows for comments and online discussions. The website is aimed at adolescents aged 13 to 17 and is pre-moderated with each comment and upload being monitored and approved before becoming available for public viewing.

The Control Condition: The two control condition websites that were chosen, 'ABC3' and 'The Fix', included features that would engage young people and bore similarities to the Bite Back website (i.e.: games and/or activities). 'ABC3' introduces young viewers to news, comedy, drama, music, sports and nature [37]. 'The Fix' engages youth in popular media news, music and videos [38]. Similar to Bite Back, both control websites are multi-component, self-guided, youth-oriented and Australian-based and each has the option of contributing personal pieces of work, opinions and stories to the website. Neither of the control websites delivers PP or information about wellbeing.

Study Procedures
The study was advertised as the "How Do You View the World Study: an investigation into how websites impact on the way young people think, react and interact with the world". It was important to conceal the clinical focus of this study for two reasons. Firstly, to ensure that control participants did not use the Bite Back website, and secondly, to minimize any expectancy effects."
12a) CONSORT: Statistical methods used to compare groups for primary and secondary outcomes
A per protocol analysis was used.

Non-parametric analysis was used due to violations of assumptions: "To analyse the efficacy of the program, a series of two-tailed Wilcoxon Signed-Rank tests were conducted to measure differences in psychopathology and wellbeing scores before and after the intervention. Non-parametric tests were employed because of violations of the normality assumption for almost all group cells (condition; condition x frequency of site visits and; condition x length of site visits). Only the post-intervention scores on the SWEMWBS did not demonstrate violations of normality. All violations were due to skew in the data that would be expected in a non-clinical population (i.e. low scores on the DASS-21 and high scores on the SWEMWBS)."
12a-i) Imputation techniques to deal with attrition / missing values
A per protocol analysis was used and as such, non-compliant and non-completers were excluded. "Non-compliant subjects and non-completers were excluded from the analysis."
12b) CONSORT: Methods for additional analyses, such as subgroup analyses and adjusted analyses
"Participants were divided into two groups based on the frequency of visits to their allocated website: low frequency (less than 3 site visits per week) and high frequency (3 or more site visits per week). There were no significant baseline differences in DASS-21 subscales or SWEMWBS scores across conditions for frequency of site visits.

Participants were also divided into two groups based on the amount of time they spent on their assigned website: 'low' (less than 30 minutes per week) and 'high' (30 minutes or more per week). There were no significant baseline differences in DASS-21 subscales or SWEMWBS scores across conditions for duration of site visits."

RESULTS

13a) CONSORT: For each group, the numbers of participants who were randomly assigned, received intended treatment, and were analysed for the primary outcome
"Of the 695 participants who expressed interest in the study, 235 met inclusion criteria. The 235 who me the inclusion criteria were allocated to either the Bite Back or control condition and completed baseline questionnaires. After the six-week trial period, 167 participants remained in the study and completed the follow-up questionnaires. A further 13 participants in the intervention condition were deemed to be non-compliant as they reported using the incorrect website (see Figure 1) as per protocol [48, 49]. Non-compliant subjects and non-completers were excluded from the analysis."
13b) CONSORT: For each group, losses and exclusions after randomisation, together with reasons
"Of the 695 participants who expressed interest in the study, 235 met inclusion criteria. The 235 who me the inclusion criteria were allocated to either the Bite Back or control condition and completed baseline questionnaires. After the six-week trial period, 167 participants remained in the study and completed the follow-up questionnaires. A further 13 participants in the intervention condition were deemed to be non-compliant as they reported using the incorrect website (see Figure 1) as per protocol [48, 49]. Non-compliant subjects and non-completers were excluded from the analysis."

This is further illustrated in the CONSORT diagram, included in the article.
13b-i) Attrition diagram

14a) CONSORT: Dates defining the periods of recruitment and follow-up
"Following baseline assessment, an email was sent to participants which included a link to their allocated website and instructions on how to use it "however and whenever" they wanted over the next 6 weeks, but "for at least an hour a week". Both Bite Back and control participants received reminder emails once a week to encourage ongoing usage of and engagement with the websites. Six weeks from their date of commencement, participants were emailed the post-intervention questionnaire battery and told that they no longer needed to access the website each week. Those who completed these questionnaires were emailed a $20 voucher from a digital media outlet."
14a-i) Indicate if critical "secular events" fell into the study period

14b) CONSORT: Why the trial ended or was stopped (early)
The trial stopped when sufficient participants had entered the study as per our power calculations.
15) CONSORT: A table showing baseline demographic and clinical characteristics for each group
Our article has a table (Table 1) that describes the demographics of each group (age, sex, and baseline scores on the measures used).
15-i) Report demographics associated with digital divide issues
"One-hundred and fifty-four participants comprised the final sample of whom one-hundred and four were female (67.5%) with a mean age of 15.4 (SD = 1.7) years. At baseline, both Bite Back and control condition participants were equivalent in mean age, gender distribution, and mean scores on the DASS-21 Depression, Anxiety and Stress subscales and on the SWEMWBS (see Table 1)."

Our article has a table (Table 1) that describes the demographics of each group (age, sex, and baseline scores on the measures used).
16a) CONSORT: For each group, number of participants (denominator) included in each analysis and whether the analysis was by original assigned groups
16-i) Report multiple “denominators” and provide definitions
Many numbers are provided in tables and figures. Please see the relevant tables and figures mentioned in the copy-paste text below.

"Of the 695 participants who expressed interest in the study, 235 met inclusion criteria. The 235 who me the inclusion criteria were allocated to either the Bite Back or control condition and completed baseline questionnaires. After the six-week trial period, 167 participants remained in the study and completed the follow-up questionnaires. A further 13 participants in the intervention condition were deemed to be non-compliant as they reported using the incorrect website (see Figure 1) as per protocol [48, 49]. Non-compliant subjects and non-completers were excluded from the analysis."

"One-hundred and fifty-four participants comprised the final sample of whom one-hundred and four were female (67.5%) with a mean age of 15.4 (SD = 1.7) years. At baseline, both Bite Back and control condition participants were equivalent in mean age, gender distribution, and mean scores on the DASS-21 Depression, Anxiety and Stress subscales and on the SWEMWBS (see Table 1)."

"Participants were divided into two groups based on the frequency of visits to their allocated website: low frequency (less than 3 site visits per week) and high frequency (3 or more site visits per week). There were no significant baseline differences in DASS-21 subscales or SWEMWBS scores across conditions for frequency of site visits.

Participants were also divided into two groups based on the amount of time they spent on their assigned website: ‘low’ (less than 30 minutes per week) and ‘high’ (30 minutes or more per week). There were no significant baseline differences in DASS-21 subscales or SWEMWBS scores across conditions for duration of site visits."
16-ii) Primary analysis should be intent-to-treat

17a) CONSORT: For each primary and secondary outcome, results for each group, and the estimated effect size and its precision (such as 95% confidence interval)
As mentioned previously, primary and secondary outcomes were not used due to the feasibility nature of our study. Likewise, estimated effect sizes and its precision (eg confidence intervals) were not used due to the article being focused on feasibility. Greater emphasis was given on other factors such as implementation issues and ease of use.
17a-i) Presentation of process outcomes such as metrics of use and intensity of use

17b) CONSORT: For binary outcomes, presentation of both absolute and relative effect sizes is recommended
As mentioned previously, estimated effect sizes and its precision (eg confidence intervals) were not used due to the article being focused on feasibility. Greater emphasis was given on other factors such as implementation issues and ease of use.
18) CONSORT: Results of any other analyses performed, including subgroup analyses and adjusted analyses, distinguishing pre-specified from exploratory
Due to the feasibility nature of this study, all analyses were exploratory and there were no pre-specified analyses.
18-i) Subgroup analysis of comparing only users

19) CONSORT: All important harms or unintended effects in each group
There were no reported or observed harms or unintended effects in either group.
19-i) Include privacy breaches, technical problems

19-ii) Include qualitative feedback from participants or observations from staff/researchers

DISCUSSION
20) CONSORT: Trial limitations, addressing sources of potential bias, imprecision, multiplicity of analyses
20-i) Typical limitations in ehealth trials

"Limitations of the study

Despite the encouraging results from this pilot study, methodological limitations preclude us from drawing definitive conclusions. Our study may have been affected by measurement sensitivity, ie. the psychopathology measure selected was designed for a clinical population and so floor effects could have impacted on our results. This problem may have been exacerbated by the small sample sizes in each condition making it less likely to obtain significant differences.

Furthermore, our usage data heavily relied on participants' self-report which could have been affected by memory biases and reporting biases [45]. Furthermore, adolescent samples may be likely under-report mental health symptoms [50, 51], although the anonymous nature of this study may have lessened this likelihood. Utilising corroborative evidence from teachers and parents may have improved the accuracy of our measurements of change in symptom alleviation and wellbeing and should be considered for further studies in this area.

The study gathered limited information about participants' use of Bite Back, such as content accessed, uploads to interactive activities, and time spent on the various activities. As the website was comprised of multiple components including videos, psycho-educational information, interactive exercises and community noticeboards, it is difficult to ascertain which parts of the website were instrumental to the changes observed. Information on usage patterns would have provided interesting insights how young people navigate the website and to better understanding the differential impact of its various components. Further research into this area would shed light on the way in which young people relate to specific online PP interventions.

The age distribution of our population was also an important factor in considering the results of this study. Whilst a larger percentage of under 16 year olds expressed interest in participating, fewer under 16s progressed through to actually participate in the study due to the need for under 16s to obtain parental permission. Given that our qualitative responses suggested that Bite Back may be more acceptable to the younger age group, this barrier to participation by younger users may have excluded an important segment of our target population in this trial.

Although Bite Back was developed as a preventative program for youth, this feasibility study was primarily concerned with obtaining feedback on users' enjoyment and willingness to engage in the site's activities. As such, our preliminary pilot data from this study is insufficient to demonstrate preventative effects. Further studies utilising a longer-term follow-up are necessary to examine this important aspect of preventative health in youth. "

21) CONSORT: Generalisability (external validity, applicability) of the trial findings

21-i) Generalizability to other populations

21-ii) Discuss if there were elements in the RCT that would be different in a routine application setting

22) CONSORT: Interpretation consistent with results, balancing benefits and harms, and considering other relevant evidence

22-i) Restate study questions and summarize the answers suggested by the data, starting with primary outcomes and process outcomes (use)

"This study examined the feasibility and challenges of an online, open-access PP program (Bite Back) to increase wellbeing and reduce mental health symptoms in young people. Despite difficulties maintaining high levels of adherence and low levels of attrition, positive qualitative feedback from participants indicated that adolescents enjoyed Bite Back and found it interesting and easy to use. In addition, significant improvements in symptoms of depression, stress and wellbeing scores were observed for the Bite Back condition."

22-ii) Highlight unanswered new questions, suggest future research

Other information

23) CONSORT: Registration number and name of trial registry

"Trial Registration:
ACTRN1261200057831; https://www.anzctr.org.au/Trial/Registration/TrialReview.aspx?id=362489 (Archived by Webcite at http://www.webcitation.org/6NXmjwfAy)"

24) CONSORT: Where the full trial protocol can be accessed, if available

The website used was provided in the references and is open-access. A Webcite version of the website was also created.

25) CONSORT: Sources of funding and other support (such as supply of drugs), role of funders

The funders of this study were specified in this article, in the acknowledgement section. Bite Back and this study was funded by the Federal Department of Health and Ageing.

X26-i) Comment on ethics committee approval

x26-ii) Outline informed consent procedures

X26-iii) Safety and security procedures

X27-i) State the relation of the study team towards the system being evaluated
